# Constructing Within-City Neighborhood Health Rankings in Philadelphia by Using Data From the 500 Cities Project

**DOI:** 10.5888/pcd18.200584

**Published:** 2021-05-13

**Authors:** Jessica Whitley, Jana A. Hirsch, Kari A. Moore, Steven J. Melly, Heather Rollins, Raynard Washington

**Affiliations:** 1 Philadelphia Department of Public Health, Philadelphia, Pennsylvania; 2 Urban Health Collaborative, Dornsife School of Public Health, Drexel University, Philadelphia, Pennsylvania; 3 Mecklenburg County Health Department, Charlotte, North Carolina

## Abstract

**Introduction:**

Profound geographic disparities in health exist in many US cities. Most reporting on these disparities is based on predetermined administrative districts that may not reflect true neighborhoods. We undertook a ranking project to describe health at the neighborhood level and used Philadelphia, Pennsylvania, as our case study.

**Methods:**

To create neighborhood health rankings, we first divided the city into neighborhoods according to groups of contiguous census tracts. Modeling our ranking methods and indicators on the Robert Wood Johnson Foundation County Health Rankings, we gathered census tract–level data from the Centers for Disease Control and Prevention’s 500 Cities Project and local sources and aggregated these data, as needed, to each neighborhood. We assigned composite scores and rankings for both health outcomes and health factors to each neighborhood.

**Results:**

Scores for health outcomes and health factors were highly correlated. We found clusters of neighborhoods with low rankings in Philadelphia’s northern, lower northeastern, western, and southwestern regions. We disseminated information on rankings throughout the city, including through a comprehensive webpage, public communication, and a museum exhibit.

**Conclusion:**

The Philadelphia neighborhood health rankings were designed to be accessible to people unfamiliar with public health, facilitating education on drivers of health in communities. Our methods can be used as a model for other cities to create and communicate data on within-city geographic health disparities.

**Summary**

**What is already known on this topic?**

Health rankings can facilitate understanding of health disparities across geographic place and identify areas of high need for public health intervention. However, research is limited on how to create health rankings within cities, rather than between cities.

**What is added by this report?**

We used local data from Philadelphia, Pennsylvania, and publicly available data from the Centers for Disease Control and Prevention’s 500 Cities Project to create within-city neighborhood health rankings.

**What are the implications for public health practice?**

Our methods serve as a model for other cities aiming to create and communicate data on within-city geographic health disparities.

## Introduction

Geographic health rankings are frequently used in public health to highlight disparities between communities and to identify areas of high need for public health intervention. Because rankings are comparative, they capture the notice of the news media and the public, stimulating conversation among community members and prompting them to act on public health issues. Most often these rankings compare health across cities, counties, or states. Within-city rankings, however, reveal disparities that are often obscured in rankings of larger jurisdictions. Within-city rankings allow for targeted local planning and can serve as a useful tool for communicating and addressing the needs of neighborhoods. The creation of healthy, equitable communities necessitates an understanding of the underlying drivers of health outcomes in different sectors of the population. The publicly available data set from the 500 Cities Project, a collaboration between the Robert Wood Johnson Foundation and the Centers for Disease Control and Prevention, features small-area health estimates at the census tract level and provides an opportunity to examine and compare the health of city residents at a small scale.

Philadelphia is an ideal case study for such a within-city health ranking. Despite overall progress in recent years, Philadelphia’s health still lags that of other major cities. Compared with counties containing the nation’s largest cities, Philadelphia has among the highest rates of premature death, infant and child mortality, frequent mental and physical distress, diabetes, HIV prevalence, and homicide (1). Underlying these poor health outcomes are high rates of adverse behavioral and economic determinants, including smoking, poverty, single-parent households, high housing costs, and low educational attainment (1). Poor health outcomes are not experienced equally by all neighborhoods in Philadelphia. The city contains several predominantly low-income and racially segregated neighborhoods that have persistent health disparities across an array of health indicators (2). Historically, the reporting of most key health indicators in Philadelphia has been limited to large geographic units known as “planning districts,” which have an average population of approximately 86,000, or to zip codes, whose boundaries do not match neighborhoods as they are perceived by residents. Additionally, although other ranking projects, such as the Robert Wood Johnson Foundation’s and University of Wisconsin Population Health Institute’s County Health Rankings (CHR), provide overviews of health in Philadelphia compared with health in other counties in Pennsylvania and health nationally, no rankings of health within Philadelphia exist at a small geographic scale.

Our project leveraged the 500 Cities data set, along with newly compiled local data, to create Neighborhood Health Rankings (NHR) for groups of census tracts that reflect commonly accepted neighborhood boundaries in Philadelphia. The objectives of our study were to describe our methods for creating the NHR and illustrate our model by using data from Philadelphia, Pennsylvania.

## Methods

**Figure 1:**
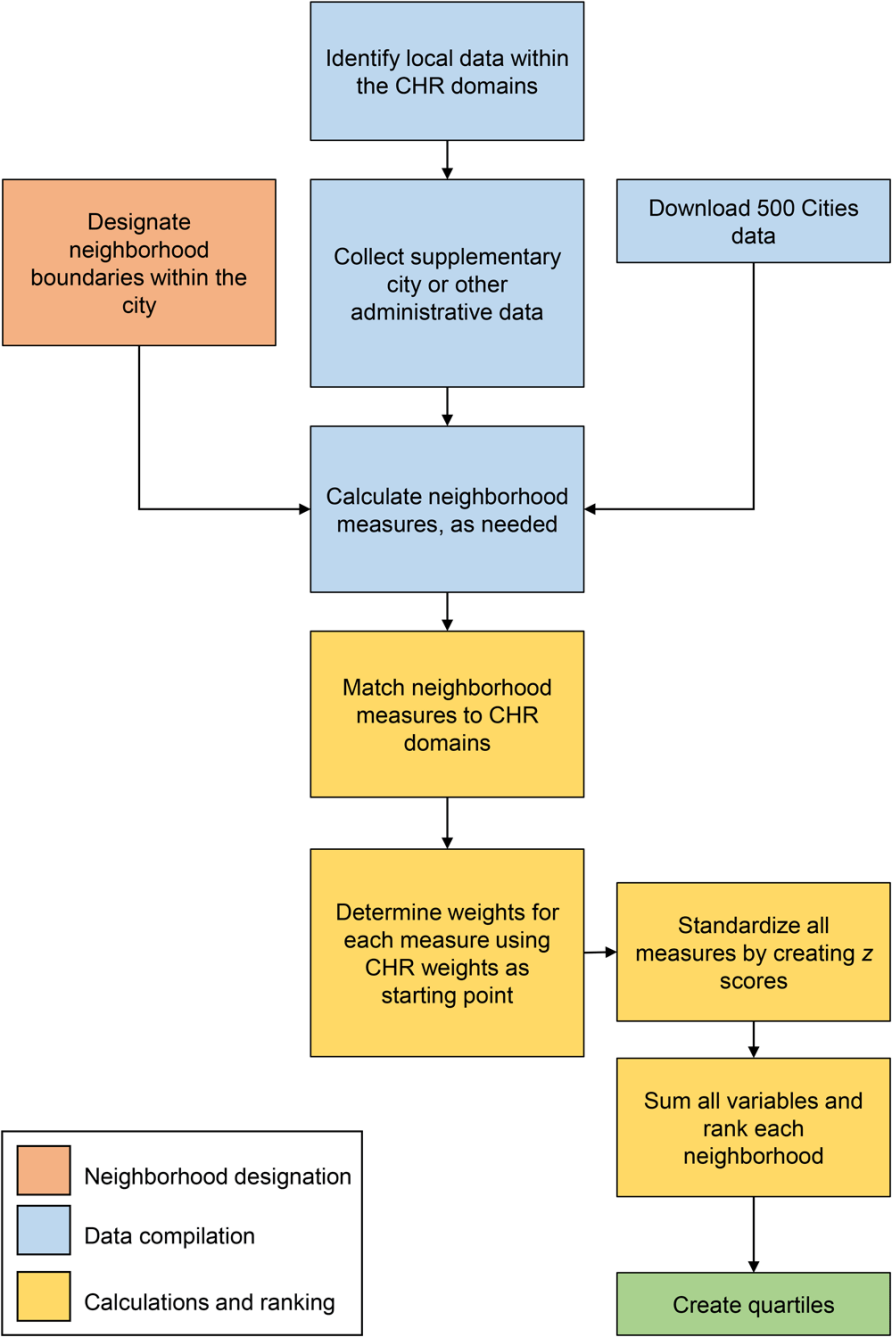
Process for creating neighborhood health rankings in Philadelphia using 500 Cities health data combined with local and administrative data. Abbreviation: CHR, County Health Rankings.

The process to create NHR was modeled after and informed by CHR (Figure 1). Our NHR were compiled from many different types of data. These data were then cleaned and standardized to construct neighborhood-level indicators. Weighted sums of these indicators were created to rank health factors and health outcomes for each neighborhood. For the Philadelphia NHR, we 1) designated neighborhood boundaries, 2) identified, collected, and processed neighborhood-level indicators that matched CHR metrics (including data from 500 Cities), and 3) calculated and constructed the neighborhood rankings.

### Defining and designating neighborhoods

Neighborhoods were designed to be small enough to represent meaningful distinctions within Philadelphia, while being large enough to have adequate data. Boundaries follow 2010 census tract lines to facilitate harmonization across data sources, including the 500 Cities data set and census data. For Philadelphia, we began with the neighborhood definitions initially created for the Southeastern Pennsylvania Household Health Survey administered by the Public Health Management Corporation (PHMC) (3). PHMC is a nonprofit public health institute that has served the greater Philadelphia area since 1972. PHMC identified 45 neighborhoods in Philadelphia according to groupings of contiguous census tracts and 2000 census tract boundaries. These neighborhoods were then aligned to 2010 census tract boundaries; we excluded census tracts designated as special land-use tracts with little or no residential population and census tracts with special characteristics such as a large park or employment area (n = 12 tracts). From these initial PHMC neighborhoods, we modified boundaries by using local knowledge. Because of its large population, we separated the Center City neighborhood into 2 neighborhoods: Center City East and Center City West. This process resulted in 46 neighborhoods with an average population of 32,978 (range, 19,503–54,167) based on American Community Survey 2012–2016 population estimates.

### Collecting data on neighborhood indicators

After creating an inventory of all CHR indicators and domains, we identified local data that aligned with similar domains. We collected city and administrative data from multiple data sources to supplement the 500 Cities data set. When identifying indicators for NHR, we focused on data that were 1) available at geographic units of census tracts or smaller to facilitate aggregation to neighborhoods and 2) generally available through public data sources or available at a city department (Table). When we could not identify data for a CHR indicator, we attempted to identify an alternate indicator for a similar construct. For example, although CHR uses alcohol-impaired driving deaths as an indicator for alcohol and drug use, these data are not available on a small geographic scale; instead, we used data on drug overdose mortality. These substitutions were not meant to be exact but were used to buttress domains with measures aligned to the original intent of the category. Therefore, NHR are an approximation rather than a direct, smaller-scale duplicate of CHR.

**Table:** Health Outcomes and Health Factors, Rankings, Indicators, and Weights in a Project to Create Within-City Neighborhood Health Rankings in Philadelphia

Domain/Broad Category	Subcategory	County Health Rankings		Neighborhood Health Rankings	
		Indicator	Weight	Indicator	Weight
**Health outcomes**					
Length of life (50%)	—	Premature death	50	Life expectancy/male	25
				Life expectancy/female	25
Quality of life (50%)	—	Poor physical health days	10	Percentage of adults aged ≥18 with poor physical health	10
	—	Poor mental health days	10	Percentage of adults aged ≥18 with poor mental health	10
	—	Low birth weight	20	Percentage of births that are low birth weight (<2,500 g)	20
	—	Poor or fair health	10	Percentage of adults aged ≥18 with asthma	1
				Percentage of adults aged ≥18 with hypertension	1
				Percentage of adults aged ≥18 with high cholesterol	1
				Percentage of adults aged ≥18 with cancer	1.5^a^
				Percentage of adults aged ≥18 with chronic kidney disease	1
				Percentage of adults aged ≥18 with chronic obstructive pulmonary disease	1
				Percentage of adults aged ≥18 with coronary heart disease	1.5^a^
				Percentage of adults aged ≥18 with diabetes	1
				Percentage of adults aged ≥18 who have had a stroke	1
**Health factors**					
Health behaviors (30%)	Tobacco use (10%)	Adult smoking	10	Percentage of adults aged ≥18 who use tobacco (current smoking)	10
	Diet and exercise (10%)	Adult obesity	5	Percentage of adults aged ≥18 with obesity	5
		Food environment index	2	Percentage of population that live beyond ½ mile from a supermarket	2
		Physical inactivity	2	Percentage of adults aged ≥18 with no leisure time physical activity	3^b^
		Access to exercise opportunities	1	Unavailable	0^b^
	Alcohol and drug use (5%)	Excessive drinking	2.5	Percentage of adults aged ≥18 who report binge drinking	2.5
		Alcohol-impaired driving deaths	2.5	Unavailable	0^c^
		Not included	0	Drug overdose mortality (per 100,000 population)	2.5^c^
	Sexual activity (5%)	Sexually transmitted infections	2.5	Unavailable	0^d^
		Teen births	2.5	Teen births (per 1,000 females aged 15–19)	5^d^
Clinical care (20%)	Access to care (10%)	Uninsured	5	Percentage with no health insurance	5
		Primary care physicians	3	Population per primary care provider (ie, number of persons per provider)	3
		Dentists	1	Unavailable	0^e^
		Mental health providers	1	Unavailable	0^e^
		Not included	0	Prenatal care access (% with inadequate care)	1^e^
		Not included	0	Have a routine medical checkup (proxy for access to care) among adults ≥18 years (%)	1^e^
	Quality of care (10%)	Preventable hospital stays	5	Percentage of adults aged ≥65 years up to date on core set of preventive services; male	2.5
				Percentage of adults aged ≥65 years up to date on core set of preventive services; female	2.5
		Diabetes monitoring	2.5	Unavailable	0^f^
		Mammography screening	2.5	Percentage of female adults aged 50–74 who have had mammography screening within the past 2 years	2.5
		Not included	0	Percentage of adults aged ≥18 with hypertension who adhere to medication	2.5^f^
Social and Economic Factors (40%)	Education (10%)	High school graduation	5	Unavailable	0^g^
		Some college	5	Percentage of people aged ≥25 who have at least some college	5
		Not included	0	Percentage of students in kindergarten through second grade who are reading at grade level (ie, reading proficiency, school quality)	5^g^
	Employment (10%)	Unemployment	10	Percentage of people aged ≥16 in the labor force who are unemployed	10
	Income (10%)	Children in poverty	7.5	Percentage of children aged <18 y living below the poverty level	7.5
		Income inequality	2.5	Income inequality: Index of Concentration at the Extremes	2.5
	Family and social support (5%)^g^	Children in single-parent households	2.5	Percentage of households that have a single parent (male or female)	5^h^
		Social associations	2.5	Unavailable	0^h^
	Community safety (5%)	Violent crime	2.5	Violent crime per 10,000 population, per Federal Bureau of Investigation	1.5^i^
		Injury deaths	2.5	Unintentional injury mortality rate (per 100,000 population)	2.5
		Not included	0	Homicides per 10,000 population	1^i^
Physical Environment (10%)^j^	Air and water quality (5%)^i^	Air pollution — particulate matter	2.5	Unavailable	0^j^
		Drinking water violations	2.5	Unavailable	0^j^
	Housing and transit (5%)^i^	Severe housing problems	2	Housing code violations: All violations recorded by the Philadelphia Department of Licenses and Inspections per 1,000 housing units	2
		Driving alone to work	2	Percentage of workers aged ≥16 who drive alone to work	2
		Long commute — driving alone	1	Percentage of workers aged ≥16 who commute ≥60 min	2
		Not included	0	Walkability (Walk Score weighted by land area)	2^j^
		Not included	0	No. of residential parcels likely to have vacant buildings divided by total no. of residential parcels	2^j^

a Cancer and heart disease were given more weight when poor or fair health weighting was distributed because they are the top 2 causes of death in the US.

b Because our indicators did not include access to physical activity facilities, we gave additional weight to physical activity itself (ie, the 1% from access is added to the 2% for physical activity to equal 3%).

c Because we did not have data on alcohol-impaired driving deaths and we wanted to capture data on alcohol and drug use, we used data on drug overdose deaths.

d Because we did not have data on rates of sexually transmitted infections, we used data on teen pregnancy to capture all weight for the sexual activity category.

e Data on dentists and mental health providers were not available, so we used data on prenatal care access and access to care among adults.

f Because we did not have data on diabetes monitoring, we used data on hypertension control as an indicator of chronic disease management.

g We used reading performance to represent school quality, because high school graduation rates are not as salient as other indicators at the neighborhood level in Philadelphia, where most students do not attend high school in the neighborhood where they reside (4).

h We were unable to obtain data on social associations, so we used data on single-parent households to represent all family and social support. The broad category should be renamed to “Family Support” or incorporated into a different category of socioeconomic status.

i To include homicides we took some weight away from violent crimes (because homicides are closer to violent crimes than to injury deaths).

j Air and water metrics were unavailable at a small spatial scale. As such, we removed subcategories in the physical environment section. The 5% for air and water were reallocated so that each of our 5 indicators in physical environment were equally weighted.

### 500 Cities data set

Information on health outcomes (asthma, hypertension, high cholesterol, cancer, chronic kidney disease, chronic obstructive pulmonary disease, coronary heart disease, diabetes, stroke, poor mental health, and poor physical health), prevention (hypertension medication adherence, mammography, older adults being up to date on a set of preventive services, routine medical checkup), and unhealthy behaviors (obesity, cigarette smoking, binge drinking, no leisure-time physical activity) was obtained from the Center for Disease Control and Prevention’s 500 Cities Project (5) for 2013 and 2014. The 500 Cities Project derived small-area estimates for census tracts from multilevel statistical models that used a poststratification approach (6,7). In December 2020, the 500 Cities Project was replaced by the PLACES project (8), which provides model-based population-level analysis and community estimates to all counties, places (incorporated and census-designated places), census tracts, and zip code tabulation areas in the US.

### Supplemental city and administrative data

We obtained vital statistics from birth and mortality records for 2016 from the Pennsylvania Department of Health, Bureau of Health Statistics and Research. We used these data to estimate life expectancy, percentage of births that were low birth weight, percentage of births with inadequate prenatal care, drug overdose mortality rates, teenage birth rates, and unintentional injury death rates. We obtained data on low levels of food access at the census tract level from the US Department of Agriculture Food Access Atlas (9). We obtained data on health insurance status, educational attainment, unemployment, childhood poverty, income, single-parent households, and commuting from the American Community Survey 5-year aggregated data for years 2012–2016 (10) at the census tract level. We obtained data on the ratio of primary care providers to population from the Leonard Davis Institute of Health Economics at the University of Pennsylvania (11). We obtained data on reading proficiency, measured as the percentage of students in kindergarten through grade 2 who are reading at grade level, from the School District of Philadelphia for K–8 schools (12). To link schools to neighborhoods, we obtained data on school locations from Pennsylvania Spatial Data Access (PASDA) (13); 14 charter schools with missing data were georeferenced to street addresses by using ArcGIS Pro version 2.0 (Esri) and the 2016 version of ArcGIS Business Analyst (Esri) address locator. We obtained data on violent crime and homicide rates in 2017 from the Philadelphia Police Department’s Incident Transmittal (INCT) system (14) available from OpenDataPhilly (15). The Philadelphia Department of Licenses and Inspections provides records of housing code violations on OpenDataPhilly.org (16). We used these records to calculate the total number of violations for 3 years (2015–2017) per 1,000 housing units, data for which were obtained from the American Community Survey 2012–2016. We obtained the Walk Score for each census tract from the City Health Dashboard (17) and data on vacant properties and buildings from the Philadelphia Office of Innovation and Technology (18). To investigate the effect of vacant land or buildings on health, we developed indicators of vacant land or buildings in residential areas (identified by land use of tax parcel data from the Philadelphia City Planning Commission [19]).

### Calculating and constructing neighborhood indicators

All data were processed and combined into neighborhood boundaries, as appropriate by source type, to create 1 measure per neighborhood. For example, if data were initially points (eg, schools, violent crimes), we assigned them to neighborhoods and created a summary measure. Most indicators included in NHR were available at the census tract level. Because neighborhoods are groupings of census tracts, we aggregated census tract data to neighborhoods.

Because 500 Cities data are estimates, they require different aggregation techniques than techniques used for nonmodeled data. We created weighted neighborhood estimates by first multiplying each census tract estimate by the proportion of the population of the neighborhood in that census tract and then summing these weighted census tract estimates in each neighborhood. Population weights reflect the 2010 US Census for the appropriate age/sex group. For most indicators, the age group was adults aged 18 or older. For indicators calculated within a subgroup, we used the respective subgroup population. We weighted the Walk Score by land area rather than population. In constructing these weighted estimates, we made the following assumptions: 1) census tracts are independent from one another, 2) the estimates follow a normal distribution on either the rate or logit scale, 3) estimates created in 500 Cities are unbiased estimates of true census tract–level rates for each indicator, and 4) rates reflect actual race/age distributions in a given census tract.

### Determining weights for each indicator

We matched indicators in NHR to indicators in CHR to designate weights (Table). We determined weights for CHR by using 5 primary methods: 1) historical perspective, 2) literature review, 3) weighting schemes used by other health rankings, 4) analytic approach, and 5) pragmatic approach (community member engagement) (20,21). Guided by CHR weights, we assigned weights to each NHR indicator (Table). We often matched functionally similar indicators. When we had more NHR indicators than CHR indicators in a domain, we assigned weights proportionally. For example, fair or poor health had a weight of 10% in CHR, which we distributed across 9 health outcome indicators in NHR. When NHR had missing indicators or fewer indicators than CHR, we distributed weight to variables in that subcategory or a broader category, and when necessary, we replaced CHR variables with similar variables. For example, CHR had a 2.5% weight for diabetes monitoring, but because we did not have data on diabetes monitoring, we distributed the weight such that NHR had 2.5% weight for hypertension medication adherence among adults diagnosed with hypertension; both are measures of chronic disease control.

### Constructing neighborhood rankings

We constructed rankings by using methods parallel to methods used in CHR: 1) standardize each indicator, 2) truncate any neighborhood indicator derived from a small sample size, 3) reverse code indicators so higher scores indicate poorer health, and 4) create a weighted sum of all indicators (21).

Indicators were standardized to the average (SD) of neighborhoods in Philadelphia (ie, *z* scores). If city boundaries covered multiple counties, we calculated *z* scores for each neighborhood in each county (standardized to the county average). For indicators where the denominator for the neighborhood indicator was 2,000 or less, we truncated any *z* score that was less than −3.0 or more than 3.0 to −3.0 or 3.0, respectively. We performed reverse coding by multiplying *z* scores by −1 for the following indicators: life expectancy (male and female), having a routine medical checkup, adults aged 65 or older being up to date on a core set of preventive services (male and female), mammography screening among women aged 50 to 74, hypertension medication adherence among adults aged 18 or older who have been diagnosed with hypertension, reading proficiency, having some college education or more, income inequality, and Walk Score. We calculated a weighted composite by multiplying each *z* score by using weights (Table) and summing. We created separate composite scores for health outcomes and health factors. We tested the correlation between the composite scores using the Pearson correlation coefficient.

We used ArcGISPro version 2.6 (Esri), R version 3.6.0 (R Foundation), and SAS version 9.4 (SAS Institute Inc) for all calculations and analyses.

### Communication and dissemination

We sorted all rankings from 1 (lowest, or best health) to 46 (highest, or worst health) in Philadelphia, corresponding to the number of neighborhoods in Philadelphia. As in CHR, NHR do not indicate significant differences between neighborhoods.

It is critical that communication of rankings does not stigmatize or lead to unintended consequences for neighborhoods or residents. Thus, to de-emphasize the differences between neighborhood ranks, we grouped neighborhoods into quartiles according to their health outcomes and health factors ranks separately. The top quartile includes the healthiest 25% of neighborhoods, and the bottom quartile includes the least healthy 25% of neighborhoods. We mapped all rankings for neighborhood health outcomes and health factors by quartile.

To facilitate use of the rankings, our dissemination strategy included a full report with neighborhood-specific information and an interactive webpage for exploring rankings in and across the city (phillyhealthrankings.org). Both the report and webpage were designed to be understandable to residents and featured visual cues to aid in understanding the data.

## Results

**Figure 2:**
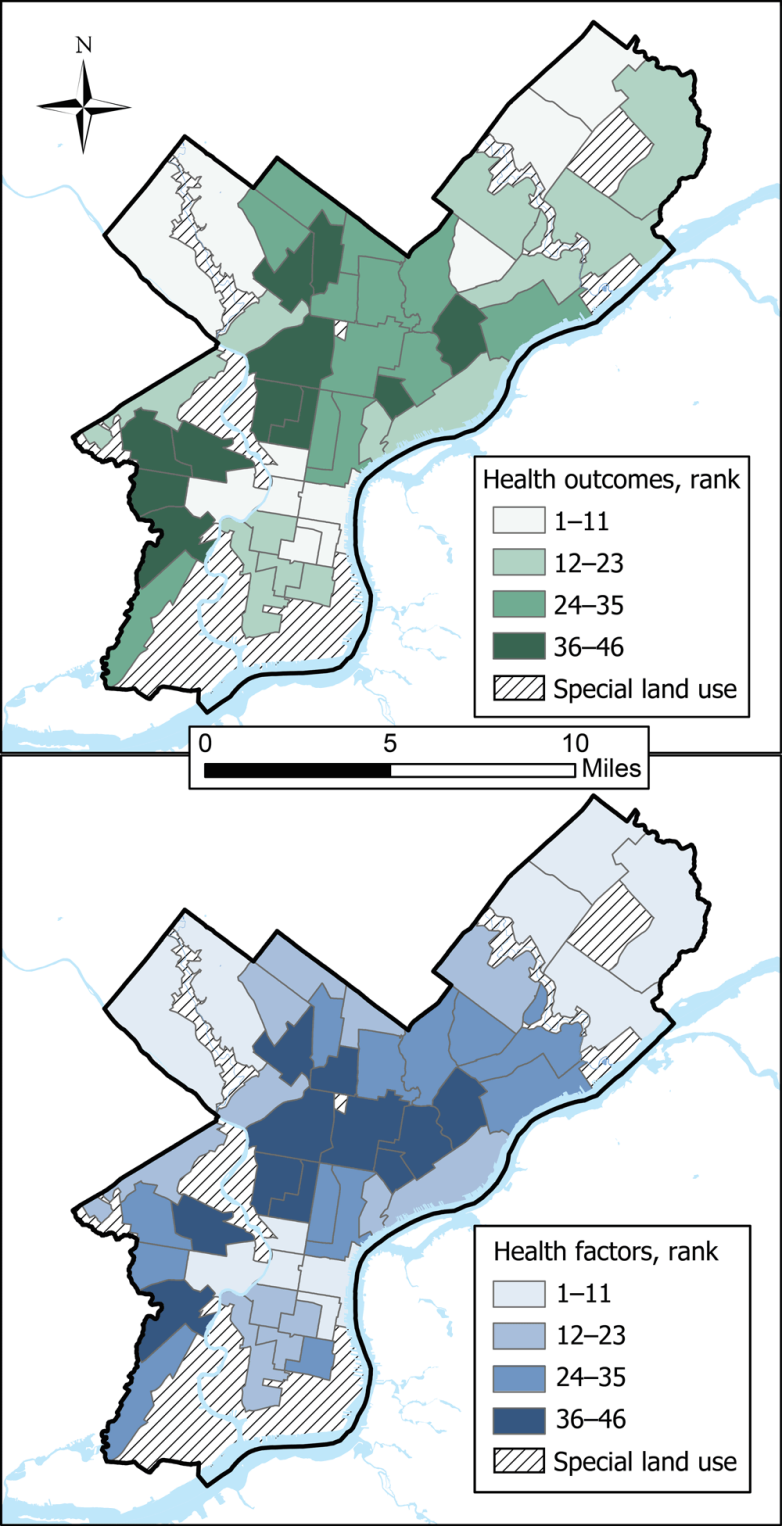
Quartiles of neighborhood health rankings in Philadelphia for health outcomes and health factors. The higher the rank, the worse the health. Diagonal hatching indicates special land-use tracts that were excluded from rankings.

**Figure 3:**
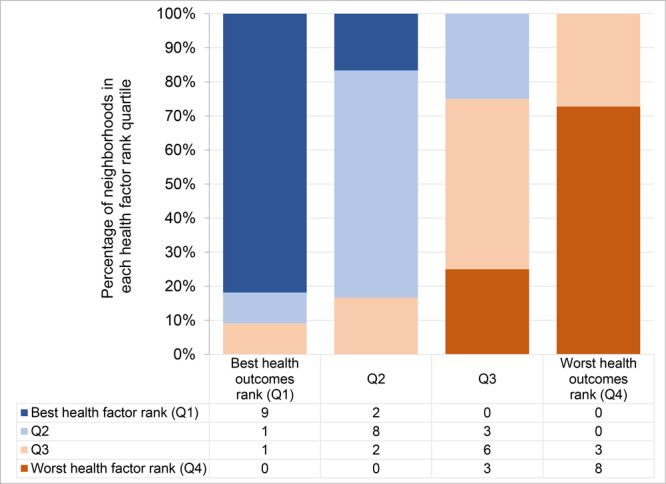
Distribution of health factor rank quartiles within health outcome rank quartiles. Numbers in chart are counts of neighborhoods in the corresponding category of both health factor and health outcome rank quartile. Q, quartile.

Poor health rankings were concentrated in Philadelphia’s northern, lower northeastern, western, and southwestern regions (Figure 2). Health outcomes and health factors scores were highly correlated (Pearson *r* = 0.90), and rankings for these 2 factors were highly correlated (Pearson *r* = 0.91). No neighborhood in the best-ranked health outcome quartile was in the worse-ranked health factor quartile and vice versa (Figure 3).

**Table:** 

Rank		Health Factor Rank				
		No. (%) of neighborhoods in Q1 (Best)	No. (%) of neighborhoods in Q2	No. (%) of neighborhoods in Q3	No. (%) of neighborhoods in Q4 (Worst)	No. of neighborhoods in all quartiles (n = 46)
**Health Factors Rank**	**No. (%) of neighborhoods in Q1 (Best)**	9 (81.8)	1 (8.3)	1 (8.3)	0	11 (100.0)
	**No. (%) of neighborhoods in Q2**	2 (18.2)	8 (66.7)	2 (16.7)	0	12 (100.0)
	**No. (%) of neighborhoods in Q3**	0	3 (25.0)	6 (50.0)	3 (27.3)	12 (100.0)
	**No. (%) of neighborhoods in Q4 (Worst)**	0	0	3 (25.0)	8 (72.7)	11 (100.0)

## Discussion

Our study outlines a novel way to use 500 Cities data in combination with local data to create within-city neighborhood health rankings. Our ranking system is based on a flexible framework that can be adapted to the availability of local data and the health factors or health outcomes most relevant to a particular population or city. Health disparities exist universally. Developing neighborhood health rankings can aid in identifying and confronting these disparities to promote health equity. Our ranking system serves as a guide for other jurisdictions looking to undertake similar projects that use the 500 Cities Project, PLACES, or other small-area data to investigate health locally. Given that the PLACES project (8) now provides wide geographic coverage across the nation, our method can be applied to create health rankings and understand drivers of health disparities more broadly than before.

Cities aiming to use our methodology should note 2 key considerations. First, the terms “community” and “neighborhood” are difficult to define and operationalize, despite theoretical work in sociology, anthropology, geography, social work, urban studies, and health research (22). As such, drawing lines between neighborhoods is often highly debated. We used and recommend the following steps for creating neighborhoods 1) scan city and local data sets for existing neighborhood boundaries (eg, planning districts, survey boundaries, neighborhood geographies, real estate boundaries), 2) designate boundaries that align with census tracts to facilitate harmonization of geographic boundaries from different data sources, 3) incorporate local knowledge from government or local agencies to finalize neighborhood designations, and 4) ensure that neighborhoods are of sufficient sample size. For aligning neighborhoods with health data, boundaries used by health agencies could be considered, where possible. Nonetheless, neighborhood boundaries may still mismatch resident perspectives and may not be stable over time if social, cultural, economic, or physical features shift. Second, available health measures for individual cities may differ from the measures used by CHR and our NHR. Cities replicating our method may need to make similar substitutions to those we made. As mentioned, these substitutions do not need to be exact, if they align to the original intent of the category. 

Unsurprisingly, areas in Philadelphia that had poor health factor rankings also had poor health outcome rankings. This finding is consistent with evidence on the social determinants of health and the association of neighborhood characteristics with health outcomes and disparities (23–25). These low-ranking collections of neighborhoods were largely in areas with higher proportions of non-White residents, who were victims of redlining beginning in the 1930s (26,27). These areas continue to feel the effects of the disinvestment and segregation, as evidenced by their poorer health rankings. The 2018 County Health Rankings Key Findings Report noted similar patterns nationwide, with many of their identified health gaps resulting from lack of opportunity and structural obstacles to good health in certain geographic areas (28). Philadelphia neighborhoods with low health outcome rankings in our study had the highest rates of child poverty, which is strongly tied to adverse health outcomes in childhood and beyond (29,30). Similarly, many of the Philadelphia neighborhoods in our study with low health factor and outcome rankings were more adversely affected than neighborhoods with better rankings by the COVID-19 pandemic, which began after completion of this project. As a result, the Black Doctors COVID-19 Consortium (www.blackdoctorsconsortium.com) focused testing and vaccination efforts on residents from specific zip codes to address disparities.

Careful and intentional dissemination of small-scale neighborhood rankings is important. It was critical that poor rankings did not create additional stigma or negative consequences for neighborhoods or residents. Our dissemination strategy emphasized NHR as a resource for Philadelphia residents to understand and act on factors affecting health in their neighborhoods, rather than a comparison of one neighborhood with another. This strategy includes deemphasizing small differences in rank (eg, using quartiles) and shaping new media narratives on connections between health factors and outcomes, rather than “best” and “worst” ranked neighborhoods. These strategies have been successful, as NHR are being used in various forms throughout Philadelphia. Aspects of the rankings were incorporated into an exhibit on heart health at a local science museum. Additionally, we have seen an interest in the geographic boundaries from local researchers and city agencies, suggesting a desire for place-based research and interventions at a meaningful geographic level. NHR have also spurred conversations among residents about health issues prevalent in their neighborhoods and structural challenges driving poor health outcomes. Other cities creating their own neighborhood health rankings should recognize potential unintended consequences and prepare a dissemination approach with a consistent messaging strategy.

Our method is one of the first attempts to use 500 Cities data for small-scale, within-city descriptions of health factors and health outcomes. It capitalizes on these data and combines them with a vast array of local data. Our methods emphasized realistic neighborhood boundaries and sound statistical methods. However, our methods have several limitations. First, several caveats exist for modeled data, such as data from the 500 Cities Project or PLACES. Because these 2 products are modeled in part by county-level data, differences across neighborhoods in cities that straddle multiple counties may reflect statistical artifacts rather than true differences. This issue can be addressed by using alternative methods of small-scale estimation that borrow across space and time (31), but use of these methods would limit the application of rankings to places with the necessary individual survey data available to create these alternative small-scale estimates. Ultimately, the complications posed by a city spanning multiple counties were not a concern in Philadelphia, where the county and city share boundaries. Similarly, researchers and practitioners should be cautious of spurious associations related to factors included in the modeling of these small-scale estimates, including age, sex, race/ethnicity, and poverty. Furthermore, because data are modeled, they may not be suitable for longitudinal comparisons. Second, NHR are an approximation that do not perfectly align with CHR (eg, information on air or water quality were not available at a subcity level with reliable accuracy). Third, neighborhood boundaries, although more realistic than previously used administrative boundaries, may not represent all geographies understood by residents. Instead, we balanced adequate data, scale of neighborhood, and generally accepted boundaries. Fourth, we produced rankings at a single point in time. We do not yet know how they will be analyzed, communicated, or interpreted should we repeat the process. Related, our neighborhood boundaries represent meaningful neighborhoods today, but they may not be relevant in the future as neighborhood demographic characteristics and social factors change. Finally, although our small-scale estimates of health provide insight into within-city disparities, disparities in health factors or outcomes likely still exist within neighborhoods, especially in neighborhoods that have heterogeneous population, environmental, or economic characteristics.

Ultimately, the creation of NHR highlighted localized pockets of high need for public health intervention in Philadelphia and revealed specific, differing barriers to health throughout the city. This knowledge facilitates targeted policies or programs and encourages more equitably allocated resources. Thus, NHR serve as a monitoring tool of Philadelphia’s health at a meaningful geographic level to improve health in our communities most at risk for adverse health outcomes. We anticipate that application of this method will similarly help facilitate identification of need, modification of policies and programs, and meaningful monitoring for resident health and disparities in other US cities.
